# Occupational Health and Safety in the Turkish Fisheries and Aquaculture; a Statistical Evaluation on a Neglected Industry

**DOI:** 10.1016/j.shaw.2023.07.004

**Published:** 2023-07-28

**Authors:** Ozan Soykan

**Affiliations:** Ege University, Fisheries Faculty, Bornova, İzmir, Turkey

**Keywords:** Aquaculture, Fisheries, Injury, Occupational safety

## Abstract

**Background:**

Fisheries and aquaculture are statistically acknowledged to be among the most dangerous occupations. Yet, industrial safety and health precautions against occupational accidents within the sector are not sufficiently implemented in many parts of the world. The present study aims to provide a quantified overview of work accident statistics in the Turkish fisheries and aquaculture industry.

**Methods:**

This article presents an overview of reported injuries and fatalities in the Turkish fisheries and aquaculture industries from 2006 to 2020. Incident, permanent incapacity, and fatality rates were calculated, and the difference between fisheries and aquaculture was statistically examined.

**Results:**

The overall incident, permanent incapacity, and fatality rates were 449.4, 4.7, and 5.7 per 100,000 worker years, respectively, over the 15-year period. With these fatality rates, fisheries and aquaculture are two of the industries with the highest fatality rates among comparable industries in Turkey. Incident rates in fisheries and aquaculture indicated that aquaculture work is more dangerous and risky. The data set includes 25 fatalities and 22 permanent incapacity cases over 15 years and shows an increase in fatality rates and occupational accidents in the last 8 years.

**Conclusion:**

present study showed that the quality of data and reporting in the Turkish fisheries and aquaculture industries including occupational illnesses, must be improved in order to be more preventative and to develop efficient safety management in the sector. Incentives for providing thorough data on occupational incidents must be enhanced to improve occupational safety awareness in Turkish fisheries and aquaculture.

## 1 Introduction

Occupational health and safety (OHS) is a branch of multi-disciplinary science focusing on improving workplace health and safety standards. The aims of OHS issues are to protect workers against occupational accidents and illnesses with a proactive approach, provide safety at the work place, and increase the quality and quantity of production [1]. Although every occupation has health or safety risks associated with it, the traditional background of fisheries and aquaculture makes it special and more complex due to the great number of mobile workers, significant health and safety issues, and variety of working conditions and environments [2], which are often dangerous and result in high fatality rates in comparison to other sectors. Some of the distinctive working conditions are unpredictable and harsh weather conditions and marine environments; unstable work platforms; moving and often heavy equipment; prolonged working periods; exposures to repetitive movements; and sometimes exposures to allergens [3]. Aquaculture practices, especially offshore, are associated with many of the same hazards as commercial fishing [4]. Moreover, employees of the fishing and aquaculture industries encounter similar risks with other challenging sectors (e.g. transportation), such as stress and fatigue, because of long working hours in a physically demanding position and high operating costs with reduced profit [5]. For those mentioned above, fishing is acknowledged as the most dangerous occupation in the world, with an estimated annual fatality rate of at least 80 workers lost per 100000 fishers [6]. According to the latest statistics, more than 58 million people are engaged in the fisheries (over 15 million are working full-time on board fishing vessels) and aquaculture industries worldwide, of which approximately 37% are engaged full-time, 23% part-time, and the rest are either occasional fishers or of unspecified status [7].

The Turkish fisheries and aquaculture industry consists of fisheries and aquaculture sectors, and the processing facilities provide services in the transformation from raw material to final product. While the total number of commercial fishing vessels was reported to be 18483, the total number of aquaculture facilities was 2036 in 2021 [8]. The total production of the Turkish fisheries and aquaculture industries has reached up to 785810 tons (53.6% of this amount belonged to aquaculture) and employed 48096 full-time equivalents in 2020 [8]. It was reported that almost 26% of the production has been exported, generating an economic volume of more than $ one billion. The milestone for Turkish industry in terms of health and safety was the enactment of the occupational health and safety law in 2012. Related to the law, fisheries and aquaculture are located in the category of “dangerous occupations” in the communique of occupational health and safety danger classes [9].

A limited number of comparative studies indicate that the Turkish fisheries and aquaculture industries have one of the highest incident rates for fatalities and permanent incapacity, together with mining, construction and the transportation industries [1,10]. Despite the fact that fishers and aquaculturists have one of the most dangerous professions in Turkey, only limited research efforts have been made so far towards improving occupational health and safety in this industry. It is also noteworthy that studies regarding the statistics and in-depth analysis of occupational incidents in the Turkish fisheries and aquaculture industry, such as fatalities and major injuries, are missing.

The aim of this article is to provide a quantified overview of work accident statistics in the Turkish fisheries and aquaculture industries, and contribute to an improved awareness of OHS in the field of fisheries and aquaculture. Additionally, scientific criticisms of the current legislation are emphasized in order to develop the concept of OHS in the sector. The results of the present work can also serve as a resource during risk assessments and for future studies regarding OHS in the fisheries and aquaculture industries. This is the first comprehensive study addressing the OHS of the Turkish fisheries and aquaculture industries from a statistical perspective.

## 2 Materials and methods

The data on occupational accidents (where it is also referred to as occupational injuries in some studies) and the precise number of employment in the fisheries and aquaculture industries from 2006 to 2020 presented in this article are collected from yearbooks of “work accidents and occupational diseases statistics” and “insured and work place statistics” of the Social Security Institution of the Turkish Republic (SGK) [11]. These documents are the only sources reporting occupational accidents and employment in Turkey. According to the communique on occupational health and safety danger classes of economic activities, the Turkish fisheries and aquaculture industries is divided into four sub-categories: marine fishery, freshwater fishery, marine aquaculture, and inland aquaculture [9]. All occupational injuries are subject to being reported to the SGK, regardless of how serious the injuries are, within 3 working days after the incident has happened, according to Turkish legislation [12].

### 2.1 Normalization of data

Standardization of the data is required in order to compare parameters between different industries and countries. Therefore, the incident rate, permanent incapacity incident rate, and fatality rate, which are the fundamental parameters of occupational accidents, have been calculated. All rates are based on the number of person years in the fisheries and aquaculture industries, where separate calculations for fisheries and aquaculture are also available as of the beginning of 2013 due to more detailed data reporting.

The incident rate (IR) is defined as the number of accidents (fatal + non-fatal) at work per 100,000 workers in employment. Separate incidence rates can also be calculated using the same formula by replacing the variable in the numerator [13].

$$IR=\frac{Number\;of\;accidents}{Number\;of\;employees\;in\;the\;covered\;population\;within\;a\;year}\times 100000$$

Permanent incapacity, in some literature given as “Total Permanent Disability,” is defined as the situation in which an insured person loses at least 10% of or the whole earning capacity due to an occupational injury or occupational disease, according to the Turkish legislation. If we arrange the abovementioned equation for the permanent incapacity incidence rate (IRp), then we get the equation below;

$$IRp=\frac{Number\;of\;employees\;with\;permanent\;incapacity\;statement}{Number\;of\;employees\;in\;the\;covered\;population\;within\;a\;year}\times 100000$$

*’*Fatal accident’ means an accident that leads to the death of a victim within one year of the accident. Therefore, the fatal incidence rate (IRf) can be expressed with the following formula:

$$IRf=\frac{Number\;of\;fatal\;accidents}{Number\;of\;employees\;in\;the\;covered\;population\;within\;a\;year}\times 100000$$

### 2.2 Limitations in the data material

Up until 2013, separate data on occupational injuries, permanent incapacities, and fatalities were not available for the subsectors of fisheries and aquaculture. Therefore, detailed results couldn't be given and compared before this year. Since the number of employees in the Turkish fisheries and aquaculture industries is not classified according to the sub-sectors by SGK, the number of employment is given to cover the fisheries and aquaculture industries separately. So the data on employment was presented according to two main legs of the industry (fisheries and aquaculture) in Table 2. Studies on official reporting systems indicate a high level of underreported cases of occupational accidents. It is acknowledged that workers and operators in the aquaculture industry underreport the average [14]. Since there is no possibility to cross-check the examined parameters for the Turkish fisheries and aquaculture industries, underreporting of injuries is discussed in section 4.5.

**Table 2:** Occupational accidents, permanent incapacities, and fatalities in fisheries and aquaculture subsectors from 2013 to 2020 in Turkey (the number of occupational accidents in fisheries and aquaculture was examined by the Mann–Whitney *U* test)

Year	Work type	Employment	Occupational accident	Permanent incapacity	Fatal accident	IR	IRf
2013	Fisheries	33455	55	2	1	164.4	3.0
	Aquaculture	8148	63		2	773.2	24.5
2014	Fisheries	32599	114	0	0	349.7	0.0
	Aquaculture	7582	82		0	1081.5	0.0
2015	Fisheries	31350	182	2	0	580.5	0.0
	Aquaculture	8041	118		1	1467.5	12.4
2016	Fisheries	32631	384	2	0	1176.8	0.0
	Aquaculture	8467	198		0	2338.5	0.0
2017	Fisheries	31842	28	1	0	87.9	0.0
	Aquaculture	9062	245		2	2703.6	22.1
2018	Fisheries	30878	45	5	0	145.7	0.0
	Aquaculture	9306	299		1	3213.0	10.7
2019	Fisheries	28717	42	3	1	146.3	3.5
	Aquaculture	9784	333		3	3403.5	30.7
2020	Fisheries	35540	41	2	4	115.4	11.3
	Aquaculture	12556	331		2	2636.2	15.9
Overall	Fisheries	257012	891	17	6	2766.7	17.7
	Aquaculture	72946	1669		11	17617.0	116.4

*U*-value = 13, The critical value of *U* at *p* < 0.05 = 15.

### 2.3 Statistical evaluations

In order to evaluate the quality and quantity of the accidents in the fishing and aquaculture sectors, statistical data on work accidents covering the whole work life in Turkey were calculated through the abovementioned 3 parameters and presented comparatively. The number of occupational accidents between subsectors of the fisheries and aquaculture industries was tested by Kruskal–Wallis variance analysis (*p* = 0.05). The difference in incidence rates, which was the only available parameter for comparison between fisheries and aquaculture, was examined by the non-parametric Mann–Whitney U test (*p* = 0.05).

## 3 Results

This section presents the annual occupational accidents, permanent incapacities, fatalities, and employment of the fisheries and aquaculture industries with a comparison of Turkey in general (section 3.1). Comprehensive results belonging to each subsector of fisheries and aquaculture were given in section 3.2. Finally, section 3.3 includes the influence of the legislation on the examined parameters of the Turkish fisheries and aquaculture industries.

### 3.1 Annual overview of the examined parameters

**Fig. 1:**
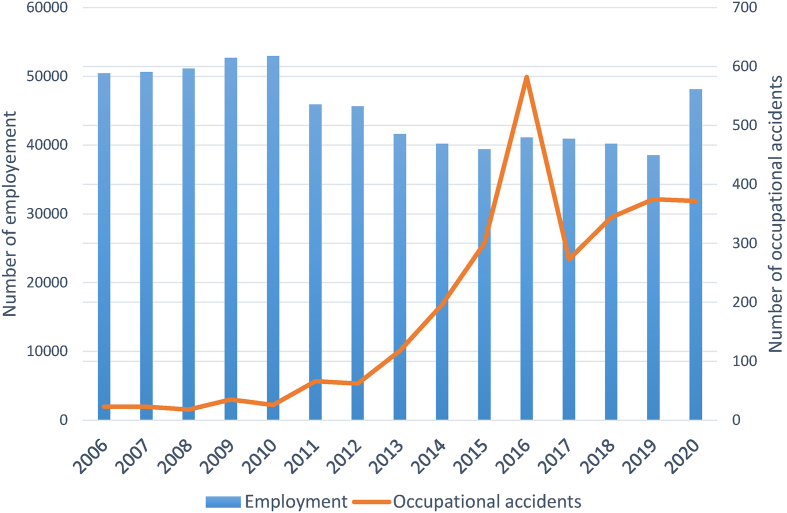
Employment and number of fatal accidents in the Turkish fisheries and aquaculture industries from 2006 to 2020.

During the 15-year study period 2006–2020, there were a total of 2813 occupational accidents that resulted in 25 fatalities and 22 permanent incapacity cases reported in the Turkish fisheries and aquaculture industries. From 2006 to 2020, employment in the Turkish fisheries and aquaculture industries ranged from 38,501 (2019) to 52,952 (2010) with an average of 45,287 ± 5126 workers per year. During 2006 and 2010, employment in the industry was more or less stable, then a relative decrease occurred in the 2011–2019 period (Fig. 1) and increased in 2020 almost up to 50,000. Despite this, the number of occupational accidents has increased, peaking in 2016. The incident rates per 100,000 workers ranged from 35.2 (2008) to 1416.1 (2016) during the fifteen years. Fatality rates in the fisheries and aquaculture industries for some years (2012, 2019 and 2020) were greater than the calculated ratios for the whole work life in Turkey (Table 1). The highest fatality rates in the fisheries and aquaculture industries were found in 2019 (10.4) and 2020 (12.5), which are significantly above the Turkish average. The overall incident, permanent incapacity, and fatality rates were 449.4, 4.7, and 5.7 per 100000 man years, respectively, over the 15-year period.

**Table 1:** Employment and some OHS parameters of Turkish labor statistics with the fisheries and aquaculture industries from 2006 to 2020

	Turkey in general							Fisheries & aquaculture						
	Employment	Occupational accidents	Permanent incapacity	Fatal accident	IR	IRp	IRf	Employment	Occupational accidents	Permanent incapacity	Fatal accident	IR	IRp	IRf
2006	14464273	79027	1953	1592	546.4	13.5	11.0	50434	23	1	0	45.6	2.0	0
2007	15114898	80602	1550	1043	533.3	10.3	6.9	50638	23	1	0	45.4	2.0	0
2008	15041267	72963	1452	865	485.1	9.7	5.8	51136	18	0	0	35.2	0.0	0
2009	15096728	64316	1668	1171	426.0	11.0	7.8	52667	35	1	1	66.5	1.9	1.9
2010	16196304	62903	1976	1444	388.4	12.2	8.9	52952	26	0	2	49.1	0	3.8
2011	17374631	69227	2093	1700	398.4	12.0	9.8	45903	66	0	3	143.8	0	6.5
2012	18352859	74871	2036	744	408.0	11.1	4.1	45622	62	2	2	135.9	4.4	4.4
2013	18886989	191389	1660	1360	1013.3	8.8	7.2	41603	118	2	3	283.6	4.8	7.2
2014	19821822	221366	1421	1626	1116.8	7.2	8.2	40181	196	0	0	487.8	0	0
2015	20773227	241547	3433	1252	1162.8	16.5	6.0	39391	300	2	1	761.6	5.1	2.5
2016	21131838	286068	4447	1405	1353.7	21.0	6.6	41098	582	2	0	1416.1	4.9	0
2017	22280463	359653	3987	1633	1614.2	17.9	7.3	40904	273	1	2	667.4	2.4	4.9
2018	22072840	430985	3773	1541	1952.6	17.1	7.0	40184	344	5	1	856.1	12.4	2.5
2019	22000964	422463	4318	1147	1920.2	19.6	5.2	38501	375	3	4	974.0	7.8	10.4
2020	23334547	384262	3118	1231	1646.8	13.4	5.3	48096	372	2	6	773.5	4.2	12.5

IR: Incident rate; IRp: Incident rate of permanent incapacity; IRf: Incident rate of fatal accident.

### 3.2 Detailed results on fisheries and aquaculture and comparison of the subsectors

**Fig. 2:**
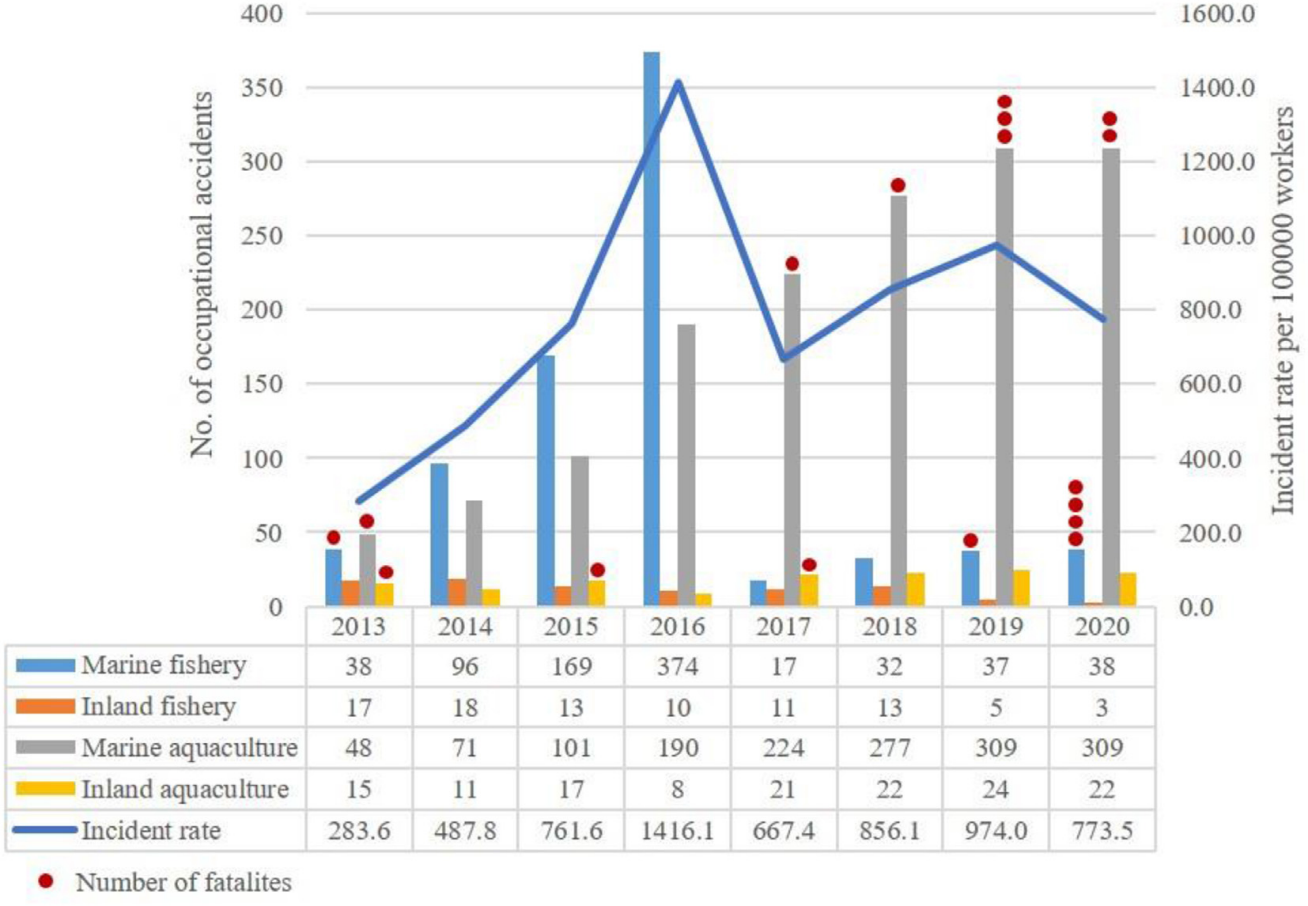
Distribution of occupational accidents and fatalities among the subsectors of the Turkish fisheries and aquaculture industries with the general incident rate from 2013 to 2020 (Red spots on the bars indicate the number of fatalities).

Results on occupational injuries, permanent incapacities, and fatalities according to the subsectors of the Turkish fisheries and aquaculture industries are presented for the period 2013–2020 because such data specific to each subsector were not available before 2013. Due to the unavailability of the number of workers separately for marine and inland fisheries and aquaculture, data were presented according to the two main legs of the industry (fisheries and aquaculture) in Table 2. The difference in the number of occupational accidents between fisheries and aquaculture was determined to be significant (Mann–Whitney U test, *U*-value = 13, critical value of *U* at *p* < 0.05 = 15). The categorized data reported to the SGK cover 2560 occupational accidents, 17 permanent incapacities, and 17 fatalities. It was determined that 11 of those fatalities occurred during aquaculture activities. Regarding the activity area, a total of 2330 injuries and 14 fatalities occurred in the marine environment, and 130 injuries and 3 fatalities happened in inland areas since 2013. During this period, the number of occupational accidents and fatalities in the aquaculture sector (1669 and 11, respectively) was higher than that in fisheries (891 and 6, respectively) (Fig. 2). Inland fishery was the only subsector without any fatalities. The overall fisheries-based incident and fatal incident rates during the 8-year study period were calculated to be 2766.7 and 17.7 per 100,000 workers, respectively. The values of these parameters are clearly higher in the field of aquaculture, where the incident rate is 17,617 and the fatal incident rate is 116.4.

In this period, a total of 1529 injuries were reported from marine aquaculture and 140 injuries from inland aquaculture. Furthermore, while the total number of marine fisheries-based injuries was 801, inland fisheries-based were reported to be 90 (Fig. 2). The greatest number of occupational accidents occurred in 2016 (*n* = 582), but no fatality was reported this year. However, 2020, which is placed within the standard error range of the average of occupational accidents between 2013 and 2020, was represented with the maximum fatalities (*n* = 6).

While the number of occupational accidents in marine aquaculture displays an increasing trend, marine fisheries peaked in 2016 and thereafter decreased sharply. The trends of inland activities were more or less stable during the 8 years in comparison to marine fisheries and aquaculture (Fig. 2). Although the number of workers in the field of aquaculture ($\bar{x}$ = 9118 ± 1465) is less than 1/3 of the marine fishers ($\bar{x}$ = 32126 ± 1862), the total number of occupational injuries in aquaculture (*n* = 1529) is almost two times that of the marine fishery (*n* = 801) between 2013 and 2020. The Kruskal–Wallis test indicated a significant difference between the mentioned activities in Fig. 2 (*KW* = 23.59, *p* = 0.0003). The greatest number of fatalities belonged to marine aquaculture, with eight deaths during 2013–2020. However, monthly injury and fatality records, types of injuries, injury mode, place of fatality, critical operations, and incidents leading to fatalities are lacking for the Turkish fisheries and aquaculture industries. In addition, although the number of occupational diseases ranged from 351 (2013) to 1208 (2007) within the Turkish industry for a 15-year period (2006–2020), no occupational disease was reported in the field of fisheries and aquaculture.

### 3.3 Effect of legislation on occupational incidents

Legislation and regulations have direct impacts on the health and safety of workers. As mentioned in the introduction section, the milestone in Turkish industry life in terms of health and safety was the enforcement of the occupational health and safety law in 2012. Evident differences can be noticed before and after the law regarding the number of employees and occupational accidents within the fisheries and aquaculture industries (Table 3). Although the average number of fishers and aquaculturists decreased after 2012 (Table 3), the number of occupational accidents, permanent incapacity cases, and fatalities increased between 2013 and 2020 (see Table 1). The differences between the two periods were also determined to be statistically significant (*p* < 0.05) except for fatality and fatal incident rates (Table 3). The probable reason of this case are further discussed in section 4.4.

**Table 3:** Comparing employment, occupational accidents, permanent incapacities, and fatal accidents in the fisheries and aquaculture industries before and after the enforcement of occupational health and safety laws in Turkey (examined by the Mann–Whitney *U* test)

	Before the law		After the law		Statistical results		
	**(2006–2012)**		**(2013–2020)**				
	Mean	Std.err	Mean	Std.err	*U* Value	*U* Value at *p* = 0.05	Difference
Employment	49907	1129	41244	1120	2	13	S
Occupational accidents	36.1	7.4	320	42.6	0	13	S
Permanent incapacity	0.7	0.2	2.1	0.5	10	13	S
Fatal accident	1.1	0.4	2.1	0.8	20.5	13	NS
IR	74.5	17.25	777.5	103.5	0	13	S
IRp	1.5	0.6	5.2	1.4	7.5	13	S
IRf	2.4	1	5	1.7	18	13	NS

∗*p* = 0.05, S: Significant; NS: Non-significant, IR: Incident rate; IRp: Incident rate of permanent incapacity; IRf: Incident rate of fatal accident

## 4 Discussion

### 4.1 Trends of occupational injuries in Turkish fisheries and aquaculture and comparisons

The initial results of this study indicate a tangible increase in the number of occupational accidents until 2017 in the fisheries and aquaculture industries. The recession in the number of occupational accidents between 2006 and 2012 may be due to underreporting and/or a lack of awareness of occupational safety. However, a significant increase after 2012 is attributable to legislation, which may have resulted in lower concealment of occupational accidents and a positive perception of occupational safety in the Turkish fisheries and aquaculture industries (further discussed in sections 4.4, 4.5). Aquaculture-based occupational accidents steadily increased and were almost two times higher than those in fisheries in 2013–2020. There may be several reasons why the number of reported cases is lower in the fishing industry. Some of them are: the number of insured employees in fisheries is less than in aquaculture, which results in less reporting. The education level of aquaculturists is considered higher than that of fishermen, enabling them to be more conscious of occupational accidents and accident reporting. Another case may be that the control and surveillance processes of OHS performed by the authority are easier in the aquaculture industries so that misreporting and/or underreporting are even harder to pronounce.

As illustrated in the present work, the overall fatality rate was 3.7 per 100,000 worker years over the 15-year period. Since 2006, Turkey's overall IR, IRp, and IRf values have generally been greater than those of fisheries and aquaculture, where fatality ratios were in favor of fisheries and aquaculture in 2012, 2019 and 2020. More importantly, fatality rates in the fisheries and aquaculture industries in 2019 and 2020 were more than two times the overall Turkish average (see Table 1). However, after 2012, detailed data provided for the subsectors of the fisheries and aquaculture industries enabled a more comprehensive discussion. The IR and IRf values of fisheries are always smaller than the overall value of Turkey, except in 2020, when the IRf of fisheries is more than 2 times the Turkish average. Comparing Table 1, Table 2 for the period between 2013 and 2020 showed that while the IR values of the aquaculture sector are generally greater than those of Turkey (except for 2013 and 2014), aquaculture's IRf values in years with fatalities are at a level of 3-4 times that of Turkey in general. Considering the inter-sectoral comparisons, significant evaluations also appear. The IR of the textile industry, one of the biggest employing industries in Turkey, was 1935 in 2016 [15], while this value was 2338.5 in the field of fisheries and aquaculture. The reported IR values of the construction industry from 2015 to 2017 as being one of the most dangerous sectors in Turkey [16] are almost at the same level as those of fisheries and aquaculture. In the present study, the overall fatal incident rate of fisheries for 2013–2020 was determined to be 17.7 per 100000 workers, which is one fifth of the Norwegian fishing fleet (87.1/100,000 workers) [17]. This significant gap may be attributable to different fishing and environmental conditions (e.g., gear type, weather, climate, sea conditions) and OHS policies and regulations between the two countries. In addition, the fishermen attendance at the insurance system may also be an important factor in the difference. On the other hand, 1669 occupational injuries have been reported in the Turkish aquaculture industry, corresponding to an overall incident rate of 17,617 for 2013–2020. It was stated that the incident rate for a 12-year period (2001–2012) in Norwegian aquaculture was 1917 per 100,000 person-years [18]. Authors also noticed that fatality rates ranged from 21.2 to 100.7 per 100,000 person-years for the aquaculture industry of Norway between 1982 and 2015 in their sister article [19]. The aquaculture based fatal incident rates of the present study fall between 3.5 and 24.5 per 100,000 person-years.

### 4.2 Quality of data and reporting in Turkish fisheries and aquaculture industries

In the fisheries and aquaculture sectors, one of the most important reasons for the high fatality rates is attributed to the distance between the working place and professional health care services, which are generally far from each other, and therefore the wounds and injuries cannot be treated rapidly. Production processes of fish and other marine resources are usually performed under unpredictable, challenging, and hostile weather conditions in a marine environment where the number of accidents and injuries is fairly high [3,20]. It is a fact that the winter months have harsher weather and sea conditions, which may result in a greater number of injuries and probably severe results. That is why the effect of seasonal changes on occupational accidents and fatalities is considered more significant in fisheries and aquaculture than in other occupations. Monthly fatality records of the Norwegian fishery where the maximum number of fatalities occurred in October, November, January, and February clearly demonstrate the effect of seasonality on fishing occupational safety [17]. Nevertheless, inadequate information on the number of fatalities and injuries occurring each calendar month for the Turkish fisheries and aquaculture industries is obstructing comparisons between studies.

The quality and quantity of historical data on occupational incidents and fatalities in an industry are of crucial importance because these are the key inputs to risk analysis, which is the fundamental approach to managing safety [21,22]. However, important parameters such as injury type, mode of injury, affected body parts, age and gender, time of year of reported injuries, place of fatalities, and location in Turkey were not given for each occupation separately. Therefore, the characteristics of occupational injuries and fatalities in the Turkish fisheries and aquaculture industries cannot be determined due to inadequate data. However, the causes of fatalities in the Norwegian fishing fleet were reported to be fire/explosion, drowning, MOB (man over board), vessel disaster, accidental, falling objects and crush impacts [17]. Aquaculture-based fatalities in Norway, from most frequent to least frequent were due to loss of vessel, blow from an object/crush, MOB, diving accident, explosion, collision, and traffic accident [19]. Besides that, information was given on the injured body areas [18], which provides data on the methods of prevention to be implemented during not only the aquaculture work but also the fisheries. The most exposed body parts were reported to be the finger, hand/wrist, hip/leg, ankle/foot and arm/shoulder. Important and lacking data in Turkish fisheries and aquaculture, such as types of injuries, injury mode, place of fatality, critical operations, and incidents leading to fatalities, should be urgently taken into consideration during reporting in order to avoid and prevent further incidents.

The incompleteness of the databases not only in Turkey but also in several other countries is one of the biggest obstacles to the promotion of OHS in the field of fisheries and aquaculture. The first step in increasing the quantity and quality of data reporting is convincing employers and employees of the need for proper data reporting in order to avoid further and more serious incidents. At this point, moral and ethical reasons can be offered to employers and workers (generally with low levels of education) in the fisheries and aquaculture industries, on the need to protect the health of the employees and support detailed data reporting. Furthermore, the data reporting system may be revised and updated according to technological innovations, and accidents that are subject to health care services may be directly transferred to social security association in order to increase the data information (types of injuries, injury mode, place of fatality, etc.).

### 4.3 The biggest gap of the system; status and awareness of occupational diseases in the Turkish fisheries and aquaculture

The concept of occupational disease in Turkey is a main subject to be significantly dwelled upon. While the number of occupational diseases ranged from 351 (2013) to 1208 (2007) in the Turkish industry for a 15-year period (2006–2020), one of the most significant points of this study is that no occupational disease has been declared so far in the field of fisheries and aquaculture. This fact does not mean that occupational diseases have never been suffered by the employees of the industry. The results of a survey study showed that joint diseases (rheumatism, meniscus tears), chronic sinusitis, and hearing loss were the most prevalent occupational diseases among fishery engineers in Turkey [23]. A current study indicated the majority of orthopedic problems were localized in the waist, back and neck area, severe pain in the joints, calcification, spine disorder and joint fluid problems among the workers in the Turkish marine fish hatcheries [24]. On the other hand, many occupational diseases in the field of aquaculture were reported based on individual case reports or systematic epidemiological studies, and those were categorized into 6 subtitles as follows: musculoskeletal disorders; dermatitis, urticarial, and skin infections; allergic respiratory disease; infections affecting other body systems; and decompression illness [25]. Although there are such references, there are no data on occupational illnesses in the field of fisheries and aquaculture, which can be explained by a lack of knowledge and awareness among the workers, employers, and government agencies and institutions. Regarding the number of anticipated occupational diseases in a given country, the study “Occupational Health” [26] is one of the leading works in the literature. The authors claimed that the number of occupational illnesses ought to be 4 to 12 per thousand workers. The mentioned ratio should be considered according to the level development in the field of occupational health in a country. In accordance with this criterion, within the years 2013–2020, it is estimated that the number of occupational diseases ought to be between 32 and 120 in the field of fisheries and aquaculture. While diagnosing and reporting occupational diseases, some deficiencies and insufficiencies have taken place, and they cause the most important problems in determining the actual result [9]. It was primarily reported that judicial/lawful process related to the determination of occupational illnesses are deprived of technical and legal devices [27]. The process takes a long time and continues insufficiently and arbitrarily. Secondly, the author stated that health care providers of “Social Security Institution” avoid responsibility while identifying occupational illnesses, and the relevant patients are dispatched to occupational disease hospitals. The researcher also claimed that these hospitals have suffered serious erosion in terms of staff and knowledge. On the contrary, the difficulty in identifying occupational illnesses and some of them being diagnosed in the long run, as well as the employees who have been changing their working area in the ongoing duration, are explained as obstacles to reporting occupational diseases [10].

### 4.4 Influence of the legislation

The results of this study strongly suggest that regulations and legislation that have been primarily designed to provide a safer work environment have direct impacts on occupational accident notification. The overall incident rate for 2006–2012, where occupational health and safety law has not been committed yet, was 74.5 per 100,000 worker years, and it was raised to more than ten times (777.5) for the period 2013– 2020. The overall permanent incapacity and fatality rates also increased from 1.5 to 5.2 and 2.4 to 5.0, respectively, during the above-mentioned periods. The difference in the examined parameters between two periods shows a considerable increase that can be commented twice. The first one is that although the number of workers in the sector has decreased since 2012, fisheries production, especially in the field of aquaculture, has increased with a positive acceleration. On a simple basis, the sector’s employees are exposed to a much higher work load, therefore, they become more vulnerable to occupational accidents, which results in a greater number of occupational incidents and fatalities. However, the second comment is as follows: After the law came into force, the reporting of occupational accidents has been controlled and inspected much more strictly than ever. Subsequently, the companies that do not obey the regulations for properly informing these accident statements are subjected to severe sanctions. These processes of sanction and inspection have increased the number of reports of occupational accidents since 2012. Although both cases may affect the increase in the number of notifications, it is considered that the second approach is more effective and realistic in view of cultural development and the present situation of occupational health and safety in Turkey.

### 4.5 Underreporting of occupational injuries

The issue of underreporting has been emphasized in comprehensive studies [18,19,25] and it is the fact that underreporting generally results in underestimating the problem's magnitude. The data set from the official statistics of SGK on fatalities is most likely more complete than that of occupational accidents. Since there is no possibility to cross-check the examined parameters for the Turkish fisheries and aquaculture industries, it is impossible to comment on the quantity of underreporting. However, according to one of the most important NGO data providers, Health and Safety Labor Watch Turkey, the number of fatalities between 2013 and 2020 covering all occupations is approximately 30% higher than the official reports. The issue of underreporting has many reasons, and among them one of the most probable is the social status of the employers. Most of the workers in the fishing and aquaculture industries are from vulnerable communities and are precariously employed. From this point on, the influence of injury reporting on personal rights is generally ignored by these workers due to a lack of awareness and unconsciousness. This is, of course, not only due to ignorance but also fear of losing the job. In addition to that, some employers put little emphasis on the health and safety of their workers and try to conceal or overlook some kinds of injuries, which also results in underreporting [18,25].

This article provides an overview of some occupational health and safety parameters in the Turkish fisheries and aquaculture industries focusing on occupational accidents, permanent incapacities, and fatalities. The data on occupational injuries and the precise number of jobs in the fisheries and aquaculture industries from 2006 to 2020 are collected from the yearbooks of “work accidents and occupational diseases statistics” and “insured and work place statistics” of the Social Security Institution of the Turkish Republic.

The data set includes 25 fatalities and 22 permanent incapacity cases over 15 years and shows an increase in fatality rates and occupational accidents in the last 8 years. Although it seems to be a negative trend, it is important that awareness of or a strict legal requirement on occupational accident reporting after 2012 has increased.

From 2013 to 2020, while the cumulative number of aquaculture-based occupational accidents and fatalities was 1669 and 11, respectively, those parameters were reported to be 891 and 6 in the field of fisheries. However, modes of fatalities, injury types, incidents by months, and injured body parts are important criteria that would be beneficial for future accident prevention but are unfortunately missing for fisheries and aquaculture in Turkey.

Underreporting is known to be one of the most important problems in the fisheries and aquaculture industries not only for injuries but also for occupational illnesses. Although the workers in the sector are vulnerable to many kinds of occupational illnesses due to the nature of the job, no case of occupational illness has been reported since 2006 in the Turkish fisheries and aquaculture industries.

Incentives for providing thorough data on occupational incidents must be enhanced to improve occupational safety awareness in Turkish fisheries and aquaculture. Obtaining data with a systematic approach would be a crucial tool for authorities and companies in order to prevent incidents, injuries, and fatalities.

## Conflicts of interest

The author has no conflicts of interest to declare.
